# The enhanced antibacterial and antibiofilm properties of titanium dioxide nanoparticles biosynthesized by multidrug-resistant *Pseudomonas aeruginosa*

**DOI:** 10.1186/s12866-024-03530-y

**Published:** 2024-10-01

**Authors:** Sayran Hamad Haji, Aryan R. Ganjo, Tola A. Faraj, Mohammed H. Fatah, Sakar B. Smail

**Affiliations:** 1 Department of Clinical Analysis, College of Pharmacy, Hawler Medical University, Erbil, Iraq; 2 Medical Research Center of, Hawler Medical University, Erbil, Iraq; 3 Department of Physiology and Microbiology, College of Medicine, Hawler Medical University, Erbil, Iraq; 4 Department of Medical Analysis, Faculty of Applied Science, Tishk International University, Erbil, Iraq; 5 Department of Medical Laboratory Technology, Kalar Technical College, Garmian Polytechnic University, Kurdistan Region, Kalar, Iraq; 6 Department of Microbiology, Par Hospital, Erbil, Iraq

**Keywords:** Anti-biofilm, Multidrug-resistant, Nanoparticles, *Pseudomonas aeruginosa*, Synergistic activity, Titanium dioxide

## Abstract

The emergence of Multidrug-resistant (MDR) bacteria are becoming a major worldwide health concern, encouraging the development effective alternatives to conventional antibiotics. The study identified *P. aeruginosa* and assessed its antimicrobial sensitivity using the Vitek-2 system. Carbapenem-resistant genes were detected through Polymerase chain reaction (PCR). MDR- *P. aeruginosa* isolates were used to biosynthesize titanium dioxide nanoparticles (TiO_2_NPs) and characterized using X-ray diffraction (XRD), Fourier-transform infrared spectroscopy (FTIR), transmission electron microscopy (TEM), field emission scanning electron microscopy (FE-SEM). A study involving 78 *P. aeruginosa* isolates revealed that 85.8% were MDR, with meropenem and amikacin showing effectiveness against 70% of the isolates. The most prevalent carbapenemase gene was *bla*_*OXA-48*_, present in 83% of the isolates. Majority of the isolates formed biofilms, and biosynthesized TiO_2_NPs were able to reduce biofilm formation by 94%. TiO_2_NPs exhibited potent antibacterial action against MDR-Gram-negative bacilli pathogens and showed synergistic activity with antibiotics, particularly piperacillin, with a significant fold increase in areas (283%). A new local strain of *P. aeruginosa*, identified as ON678251 in the World GenBank, was found capable of producing TiO_2_NPs. Our findings demonstrate the potential of biosynthesized TiO_2_NPs to manage antibiotic resistance and regulate the formation of biofilms. This presents a promising direction for the creation of novel antimicrobial agents or substitutes for use in clinical settings, particularly in the management of isolates capable of resisting multiple drugs.

**Supplementary Information:**

The online version contains supplementary material available at 10.1186/s12866-024-03530-y.

## Background

Antimicrobial resistance is a major contributor to the high rates of illness and death caused by bacterial infections worldwide [1]. *Pseudomonas aeruginosa* exhibits a diverse array of pathoadaptive traits and pathogenicity mechanisms that enhance its capacity to inhabit, survive, and proliferate in different habitats [2]. The incidence of *P. aeruginosa* infections is on the rise, particularly in hospitalized patients, and there is an emergence of multidrug-resistant strains. Carbapenems have traditionally been reserved as a last-resort treatment for controlling infections caused by MDR-*P. aeruginosa*. However, it is important to note that carbapenem-resistant *P. aeruginosa* may also exhibit resistance to other classes of antimicrobial drugs, leaving limited treatment options and resulting in high rates of illness and death, especially among hospitalized individuals and those with weakened immune systems [1]. The incidence of carbapenem-resistant *P. aeruginosa* in nosocomial infections is increasing, with rates varying from 0–60% [3]. *Pseudomonas* are known for their ability to rapidly develop resistance to commonly used antibiotics, due to the presence of multiple efflux pumps. Additionally, *P. aeruginosa* is an extremely virulent bacterium, highlighting the urgent need for new antimicrobials [4]. The production of biofilms presents a major obstacle for the host due to the frequent resistance of these biofilms to antibiotics, phagocytosis, and surfactants. Eliminating the biofilms once they have formed is challenging due to this resistance [2, 5]. Biofilms provide a protective growth form for the bacteria and are crucial for survival in healthcare environments, while also maintaining an inflammatory environment in the host [3]. The sensitivity of bacteria to antimicrobial agents varies between their planktonic and biofilm forms. Biofilm-forming microorganisms are more resistant to antibiotics compared to bacteria that grow as individual cells. The biofilm produced by *P. aeruginosa* is recognized as a major contributor to treatment failure [6].

Carbapenem resistance in *Pseudomonas aeruginosa* is frequently associated with the acquisition of particular genes that encode carbapenemases, which are enzymes capable of degrading carbapenem antibiotics. These genes are commonly found on mobile genetic components, which enables them to easily travel among various bacterial strains and species. The predominant carbapenemases detected in *P. aeruginosa* include metallo-beta-lactamases (MBLs) such as VIM, IMP, and NDM, as well as serine carbapenemases like the OXA-type enzyme. These enzymes not only provide resistance to carbapenems but also to other antibiotics, making treatment more difficult [2, 7]. The transfer of new resistance genes via horizontal gene transfer can result in resistance to the four main categories of antipseudomonal agents. These include important cephalosporins (including combinations with β-lactamases), carbapenems, aminoglycosides, and respiratory fluoroquinolones (such as ciprofloxacin, levofloxacin, and moxifloxacin), as well as colistin [3]. The emergence and widespread dissemination of acquired carbapenem hydrolyzing enzymes (carbapenemases) are significant concerns in terms of global health. These enzymes pose serious challenges for therapy and infection control, leading to increased mortality rates and longer hospital stays. Consequently, clinicians have become increasingly concerned about promptly identifying bacteria carrying these genes to mitigate or prevent this issue in healthcare facilities [8]. Therefore, there is a need for alternative strategies to combat bacterial infections and block resistance mechanisms. One such strategy is the use of metallic nanoparticles (NPs) [9]. Nanotechnology offers a promising platform for developing NPs with antimicrobial properties that can work synergistically with antibiotics against different bacteria also has anticancer properties to treat human breast cancer [10, 11]. Bacterial NP synthesis is favored among biological entities because of its large production, quick growth rate, and controllability [12]. TiO_2_NPs, in particular, have attracted significant interest due to their diverse applications and favorable properties [13]. Nanoparticles can disrupt the bacterial outer membrane, inhibit enzymes, induce changes in gene expression, and trigger pathways for cell death. They are also more stable and less toxic than traditional antibiotics, consequently, the medical sector becoming more interesting in employing these NPs as an alternative or co-antimicrobial agent [14]. Inorganic minerals like silver, gold, titanium, copper, and zinc have been explored as alternatives to antibiotics and disinfectants, with applications in preventing catheter-associated infections, inhibiting biofilm formation, and serving as drug delivery systems [15]. Antibiotics are less effective because of their poor intracellular bioavailability and non-target-specific methods of action. Additionally, resistance develops as a result of its broad use. Furthermore, using certain antibiotics can have adverse consequences like cytotoxicity. These issues can be resolved by the creation of novel treatment approaches, such as NP therapy in combination with other antimicrobial medications [16]. The combination of NPs with antibiotics in "combination therapy" has emerged as a promising approach to combat multidrug-resistant bacteria. This approach can overcome bacterial resistance mechanisms and enhance the efficacy of antibiotics [17, 18]. While antibacterial NPs will not only shield drugs from the molecular mechanisms of resistance, their combined actions with antibiotics are expected to prevent mechanisms of bacterial resistance to antibacterial agents. Instead, the delivery mechanism for NPs will confer an antibacterial action by a synergistic combination with nanoparticles, reinforcing traditional antibiotics against multidrug-resistant bacteria [19].

This study investigates MDR in *P. aeruginosa* and evaluates the antibacterial activity of biosynthesized TiO_2_NPs against MDR-Gram-negative bacteria, both alone and in combination with antibiotics, and their impact on biofilm production.

## Methods

### Bacterial isolates

The study encompassed 78 clinical samples of *P. aeruginosa* that had multidrug resistance (MDR), including resistance to carbapenem. These samples were gathered from both hospital and community settings in Erbil, which is located in Iraq's Kurdistan Region, over the period from January to September 2021. The specimens were obtained from several locations of infection, namely urine (24 specimens), sputum (16 specimens), wound swabs (8 specimens), and blood (3 specimens)). The study involved 78 patients aged 1 month to 80 years, with a median age of 40, with 60.3% being female. The antibacterial activity of biosynthesized TiO_2_NPs was evaluated against MDR Gram-negative bacilli strains from Rizgari Hospital in Iraq, including *E. Coli, Klebsiella pneumonia (K. pneumonia), Acinetobacter baumannii (A. baumannii),* and *Proteus sp.*

### Antibacterial susceptibility test

The antimicrobials that were analyzed comprised imipenem and meropenem (carbapenems), trimethoprim/sulfamethoxazole (sulfonamides), gentamicin and amikacin (aminoglycosides), ciprofloxacin and levofloxacin (quinolones), and piperacillin and piperacillin-tazobactam (penicillins), along with tigecycline (tetracycline). The susceptibility of *P. aeruginosa* isolates was evaluated using the Vitek-2 automated method, and resistance profiles were utilized to assign MDR [3]. The ATCC *P. aeruginosa* 27853 strain served as the quality control reference strain.

### Biofilm formation assay

The study employed the microtiter plate test method to identify the isolates' ability for biofilm formation [3] The isolates were incubated overnight in tubes containing 5 mL of trypticase soy broth (TSB) (Merck, Germany) at a temperature of 37°C. The expansion was subsequently reduced in a new solution at a ratio of 1 part growth to 100 parts fresh medium. Subsequently, 200 μL of the diluted cultures were introduced into 96-well polystyrene microtiter plates (Costar/USA) and subjected to incubation at 37°C for 24 h under static conditions. Parafilm was applied to the edge of the plate to inhibit evaporation while it was being incubated [2]. Subsequently, the wells were rinsed thrice with 200 μL of Phosphate Buffer Saline at a pH of 7.2 to eliminate the broth, followed by air-drying. Subsequently, the wells were treated with 200 μL of 0.1% crystal violet solution for 30 min at ambient temperature. Subsequently, the plates were rinsed with distilled water to eliminate any dye that was not attached and left to air dry. The stain that was attached was dissolved by adding 200 μL of 95% ethanol, and the optical density (OD) of the dissolved crystal violet was measured at 630 nm using an ELISA reader. Biofilm formation was deemed unfavorable when the optical densities (ODs) were below 0.12, moderately favorable when the ODs ranged from 0.12 to 0.24, and highly favorable when the ODs exceeded 0.24. The experiment was conducted on three separate occasions, and the average of the recorded numbers was reported as the outcome [20].

### Molecular analysis of carbapenemase genes

Carbapenemase genes, specifically MBLs (*bla*_*NDM*_*, bla*_*VIM*_*,* and *bla*_*IMP*_), OXA-48 (*bla*_*OXA-48*_), and KPC (*bla*_*KPC*_) genes, were screened in *P. aeruginosa* utilizing primers from earlier research [7] Carbapenemase genes, specifically MBLs (*bla*_*NDM*_*, bla*_*VIM*_*,* and *bla*_*IMP*_), OXA-48 (*bla*_*OXA-48*_), and KPC (*bla*_*KPC*_) genes, were screened in *P. aeruginosa* utilizing primers from earlier research through the utilization of polymerase chain reaction. Multiplex PCR was utilized to identify the presence of carbapenemase genes. The genomic DNA extraction process involved the utilization of a commercial extraction kit (DNAL and Scientific Cat No. GG2001) to extract the entire DNA content from bacterial cultures during the logarithmic phase. The extraction was performed according to the instructions provided by the manufacturer. The PCR amplification stages were conducted using a thermocycler machine (Techne, UK) with a total volume of 25 μl. The reaction mixture consists of 12.5 μl of Gotaq Green Master Mix (Promega/USA), 3 μl of genomic DNA, 1.5 μl of each primer, and 6.5 μl of nuclease-free water. Thermocycling conditions included a 1 min warm-up at 95 °C, 30 cycles of 30 s at 95 °C, 30 s at 55 °C, and 1 min at 72 °C, culminating in 7 min extension step at 72 °C. the DNA fragments were visualized by electrophoresis in a 2% agarose gel [4, 7].

### Biosynthesis of TiO_2_NPs

*Pseudomonas aeruginosa* cells were cultured in sterile TSB medium and incubated at 37 °C with shaking at 120 rpm for 24 h. A transformation mixture was prepared by centrifuging 50 mL of the culture broth at 8000 rpm for 10 min, and mixing 20 mL of culture supernatant with 20 mL of 0.025 M TiO_2_ (Sigma Aldrich) to achieve a 1:1 ratio. The mixture was heated in a water bath at 80 °C for 10–20 min until white deposition appeared at the bottom, indicating transformation. The solution was cooled and incubated at room temperature. After 12–48 h, distinct coalescent white clusters were observed at the bottom of the flask. The resulting suspension was centrifuged at 2000 rpm for 20 min, and the supernatant liquid was removed. The residue was washed multiple times with deionized water to remove impurities. The precipitate was dried in an oven at 40 °C for 30 min. The synthesized TiO_2_NP samples were then subjected to characterization [21].

### Characterization of TiO_2_NPs

The process of NP formation was monitored by observing the change in color of the culture supernatant. This change was verified by utilizing a dual-beam UV–visible spectrophotometer (Perkin Elmer Lambda model 35USA), to measure the peak exhibited by the TiO_2_NPs. The UV–visible spectra of 2 ml samples of the culture supernatant were measured in the wavelength range of 300–800 nm, using double distilled water as a reference [22]. The properties of the functional groups in TiO_2_NPs were analyzed using Fourier-transform infrared spectroscopy (FT-IR). The FT-IR study was conducted using the Spectrum 4600 instrument from JASCO. The analysis focused on the spectral range of 4000–400 cm − 1 with a resolution of 4 cm − 1 [23].

X-ray diffraction (XRD) measurements were conducted using the PAN analytical X’Pert PRO instrument to analyze the crystalline structure and chemical content of the TiO_2_NPs. The XRD analysis employed Cu K (k = 1.5406) radiation, with the apparatus configured at 40 mA and 40 kV. The diffraction intensities were measured within the angular range of 20°–80°, specifically in the 2θ region. TiO_2_NPs in dry powder form were placed on the XRD grid for investigation [24, 25]. The particle size distribution and morphology of the TiO_2_NPs were analyzed using advanced transmission electron microscopy (TEM) (TEM; Titan3 G2 60–300, Cs corrector: image and probe, FEI, USA) and field emission scanning electron microscopy (FESEM) (Quanta 4500). The sample for TEM examination was made by drop casting onto a grid coated with Cu TEM holey carbon and then air-dried, resulting in a thin film. An investigation using Field Emission Scanning Electron Microscopy (FESEM) was performed on a sample that was prepared on a copper grid coated with carbon. Furthermore, the chemical composition of the TiO_2_NPs was examined using energy dispersive X-ray spectroscopy (EDX) in combination with field emission scanning electron microscopy (FESEM). The specimens were processed and dehydrated on a carbon-coated copper grid [26].

### Antimicrobial activity of biosynthesized TiO_2_NPs

1. Antibiotic susceptibility testing by disc diffusion method
  The Kirby-Bauer disc diffusion assay was used to assess the susceptibility of MDR Gram-negative bacilli isolates (*E. coli, Klebsiella sp., A. baumannii, Proteus sp.* and *chosen P. aeruginosa*) to β-lactam antibiotics. The antibiotics used were ceftazidime (30 μg), cefoxitin (30 μg), piperacillin (100 μg), and imipenem (10 μg). The McFarland standard solution was used to standardize the inoculum density. A sterile swab was used to inoculate the suspension, which was then streaked on a Muller-Hinton agar (MHA) plate. Antimicrobial discs were placed on each plate after a 5-min incubation at room temperature. The plates were incubated at 37 °C for 18–24 h and the results were compared to the standard levels specified in the CLSI (2020) documentation [27].
2. The antimicrobial susceptibility of TiO2NPs alone and in combination with antibiotics
  The antibacterial activity of TiO_2_NPs was assessed using a disc diffusion assay. A suspension of MDR- *E. coli, Klebsiella sp., A. baumannii, Proteus sp.,* and *P. aeruginosa* isolates was prepared at a concentration of 1.5 × 108 CFU/mL and swabbed onto MHA plates. Plain TiO_2_NP discs were created by adding 10 µl of TiO_2_NPs (5000 µg/mL) to 6 mm diameter Whatman paper discs [28]. The effect of TiO_2_NPs in combination with antibiotics was assessed by adding 10 µl of the defined concentration of TiO_2_NPs to standard discs of ceftazidime, piperacillin, cefoxitin, and imipenem. A disc soaked in sterile distilled water was used as a negative control, and various discs were inoculated onto MHA plates and incubated at 37 °C for 24 h. The disc diffusion assay was conducted in triplicate, measuring and comparing inhibition zones with the standard antibiotic inhibition zones published by CLSI (2020) [27].
3. Evaluation of the synergistic effect of TiO2NPs with antibiotics
  The study evaluated the synergistic effect between TiO_2_NPs and antibiotics by calculating the fold increase in the diameter of the inhibition zone for each antibiotic when combined with TiO_2_NPs. The calculation used the equation: Fold increase = (B-A)/A × 100, where (A) represents the inhibition zone of the antibiotic alone and (B) represents the inhibition zone of the antibiotic combined with nanoparticles [29].

### Antibiofilm potential of TiO_2_NPs

The study used a 96-well microtiter plate method to evaluate the effectiveness of biosynthesized TiO_2_NPs in biofilm formation [3]. The plates were filled with 180 μL of MH broth and inoculated with 10 μL of an overnight culture. 10 μL of TiO_2_NPs were added to achieve a concentration ranging from 19.531 to 5000 μg/mL. The plates were incubated at 37 °C for 24 h, then washed three times with PBS (pH 7.2) to eliminate free-floating bacteria. The biofilms formed by adherent organisms were fixed with 2% sodium acetate and stained with 0.1% crystal violet dye. The excess stain was rinsed off with sterilized Millipore water, and the plates were dried. After drying, 200 μL of 95% ethanol was added to each well. The absorbance at 620 nm was measured using an ELISA reader (Multiskan® EX, Thermo Scientific, Finland), and the percentage of biofilm inhibition was calculated using an equation: % biofilm inhibition = [(1 − OD620 of cells treated with TiO_2_NPs/OD620 of non-treated control) × 100]. The experiment was conducted three times. A bacterial cell-free filtrate (10 μL) used as a positive control in the preparation of TiO_2_NPs.

### Sequencing of 16S rRNA gene

The PCR products were sent to the National Instrumentation Center for Environmental Management in Seoul, Korea for sequencing using primers PA-SS-F (GGGGGATCTTCGGACCTCA) and PA-SS-R (TCCTTAGAGTGCCCACCCG) [30]. The nucleotide sequence of the *P. aeruginosa* strain was submitted to the National Centre for Biotechnology Information (www.ncbi.nlm.nih.gov), assigned GenBank accession number ON678251. The 16S rRNA partial gene sequencing was analyzed using the Basic Local Alignment Search Tool (BLAST), which compares the sequence with other biological sequences to identify similarities with the *P. aeruginosa* isolate. Similarities between bacterial strains were aligned using the CLUSTAL W phylogenetic tree and extracted from nucleotide sequence databases. The Molecular Evolutionary Genetics Analysis (MEGA) version 11 software was utilized to construct a neighbor-joining tree.

### Analysis of statistics

The data was analyzed using GraphPad Prism and a chi-square test, with a statistically significant *p*-value of less than 0.05.

## Results and discussion

### Characteristics of the bacterial isolates and their demographics

The study analyzed 78 MDR *P. aeruginosa* isolates from various clinical specimens, finding no significant association between gender and clinical specimens, despite a significant gender-related association (*p* > 0.05) (Table 1).

**Table 1:** Epidemiological and clinical characteristics of the *Pseudomonas aeruginosa* isolates

Variables	No	%
Gender		
Male	31	39.7
Female	47	60.3
Age		
1month-20 years	4	5.1
20–40	15	19.2
40–60	19	24.3
60–80	40	51.2
Clinical specimen		
Sputum	35	44.8
Urine	18	23
Swab	15	19.2
Cerebrospinal fluid	8	10.2
Blood	2	2.5

*P*- value = 0.31

Among the discovered *P. aeruginosa* isolates, the majority (60.3%) were from females, while 39.7% were from men. The highest infection rate was observed among individuals aged 60–80 years (51.2%). This result aligns with a prior investigation conducted by Subramaniyan and Sundaram, which revealed that 31.1% of the samples were male and 68.8% were female [31]. The majority of isolates (35, 44.8%) were obtained from sputum (35, 44.8%), followed by urine samples (18, 23%), swabs (15, 19.2%), cerebrospinal fluid (8, 10.2%), and blood (2, 2.5%) (Table 1). The largest number of clinical isolates was obtained from sputum, indicating the presence of respiratory tract diseases. The findings align with the research conducted by others, who similarly observed a significant occurrence of *P. aeruginosa* in tracheal aspirates [31, 32].

### Antimicrobial resistance

The Vitek-2 automated system test indicated that all 78 *P. aeruginosa* isolates exhibited significant resistance to the antimicrobials that were evaluated. A total of 13 antibiotics belonging to seven different classes were chosen for antibiogram profiling because of their widespread usage in hospitals as medications to combat *pseudomonas* infections. The isolates shown substantial resistance to multiple tested antimicrobial drugs (*p* < 0.001) (Table 2).

**Table 2:** The antimicrobial resistance profile of *Pseudomonas aeruginosa* isolates. The results are repeated triplicate *p* < 0.001

Antibiotics Classes	Antibiotics	Resistance rates (%)
Carbapenem	Imipenem Meropenem	40 (51.2)
		26 (33.3)
Cephalosporin	Cefepime Ceftazidime Cefoxitin	57 (73)
		53 (67.9)
		54 (69.2)
Penicillin	Piperacillin Piperacillin/tazobactam	63(80.7)
		61 (78.2)
Sulfonamides	Trimethoprim/Sulfamethoxazole	49 (62.8)
Tetracycline	Tigecycline	50 (64.1)
Quinolones	Levofloxacin Ciprofloxacin	30(38.4)
		28 (35.8)
Aminoglycoside	Amikacin Gentamicin	26 (33.3)
		27 (34.6)
Resistance profile (MDR) 67 (85.8%)		

*P*-value < 0.001

The study revealed that the greatest level of resistance was observed against β-lactam antibiotics, specifically within the penicillin class [piperacillin 63 (80.7%), piperacillin/tazobactam 61 (78.2%)], cephalosporin class [cefepime 57 (73%), ceftazidime 53 (67.9%), and cefoxitin 54 (69.2%)], as well as the tetracycline class [Tigecycline 50 (64.1%)]. Table 2. These findings are consistent with prior research [1, 3, 20]. The increasing prevalence of drug resistance poses a significant challenge for treatment options, particularly with the emergence of high carbapenem resistance among Gram-negative bacilli isolates, which has been extensively reported in various countries. Consequently, carbapenem resistance has become a critical global public health crisis [33]. In the present study, the rate of imipenem resistance against MDR-*P. aeruginosa* was moderately high at 51.2% (Table 2). This emergence of such resistant isolates is a cause for concern in public health. A study in Isfahan found that out of 106 *P. aeruginosa* isolates, 62 (58.5%) were resistant to imipenem [34]. This is lower than previous reports, which reported 17.7% resistance [1]. In Iran, imipenem resistance in burn and non-burn patients was 83.2% and 57.5%, respectively [35]. Additionally, 33.3% of isolates showed resistance to meropenem, a highly effective antipseudomonal antibiotic. This finding aligns with studies in Bangladesh and Nepal [20, 36]. Resistance to other classes of antibiotics was linked to resistance against carbapenem antibiotics. Rates of resistance were found to be tigecycline (64.1%), trimethoprim/sulphamethoxazole (62.8%), levofloxacin (38.4%), ciprofloxacin (35.8%), gentamicin (34.6%), and amikacin (33.3%). Interestingly, meropenem and amikacin were effective against approximately 70% of the MDR-*P. aeruginosa* isolates. Amikacin was found to be the most effective antipseudomonal antibiotic for MDR-*P. aeruginosa* infections and a suitable treatment option for carbapenem-resistant *P. aeruginosa* isolates. Amikacin's resistance rate (33.3%) aligns with de Sousa et al.'s findings [2], but a different study reported a high resistance rate (92.4%) in burn infections caused by carbapenem-resistant *P. aeruginosa* isolates [37]. In brief, 85.8% of strains exhibited resistance to at least three classes of antimicrobials, making them MDR-*P. aeruginosa*. This result is consistent with the high rates of MDR-*P. aeruginosa* isolates in Iran, where 89.4% of the isolates were found to be MDR [38]. There is a growing occurrence of MDR-*P. aeruginosa* isolates worldwide, and this study has found higher rates than prior findings. Research has also indicated that patients infected with *P. aeruginosa* and *Acinetobacter* spp. have a significant risk of death, with mortality rates ranging from 40 to 65% [39]. The high resistance to carbapenem antibiotics is concerning from a public health perspective, as these antibiotics are essential for treating infections when lower-class antibiotics are no longer effective [2]. The higher incidence of multidrug-resistant (MDR) patterns among strains in this study may be attributed to factors such as self-medication, empirical usage, and excessive use of carbapenems and third-generation cephalosporins. Additional surveillance initiatives are required in this region to effectively tackle the escalating issue of carbapenem resistance [40].

### Biofilm production assay

The study revealed that 78.2% of 78 isolates were biofilm producers, indicating their higher resistance to antibiotics compared to free-floating cells [9].

**Fig. 1:**
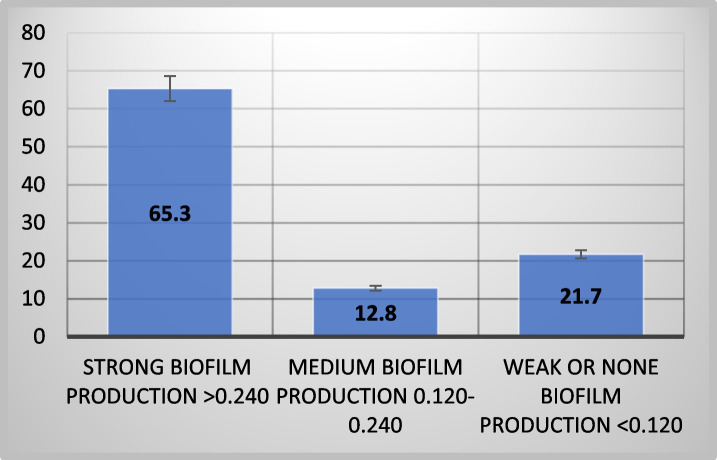
Biofilm-production capacities among *Pseudomonas aeruginosa* isolates

Figure 1 shows that most strains (65.3%) had strong biofilm formation capacity, while 12.8% and 21.7% exhibited moderate or weak biofilm-forming potential, respectively, using the tissue culture plate method.

Most pathogenic *P. aeruginosa* strains can form biofilms, making them difficult to remove from hospital environments and medical equipment. There was a significant (*p* < 0.0001) difference in biofilm production abilities between tested MDR-*P. aeruginosa* isolates. The study found that all *P. aeruginosa* strains, including clinical specimens, can form biofilms under laboratory conditions, although the extent of biofilm formation varies. This aligns with previous studies, where 19.87% of isolates were weak/non-biofilm producers, 20.86% were moderate producers, and 59.27% were strong biofilm producers [3]. In contrast, de Sousa et al. reported higher biofilm production rates, with 92% of *P. aeruginosa* isolates producing biofilms [2].

### Prevalence of carbapenemase genes

**Fig. 2:**
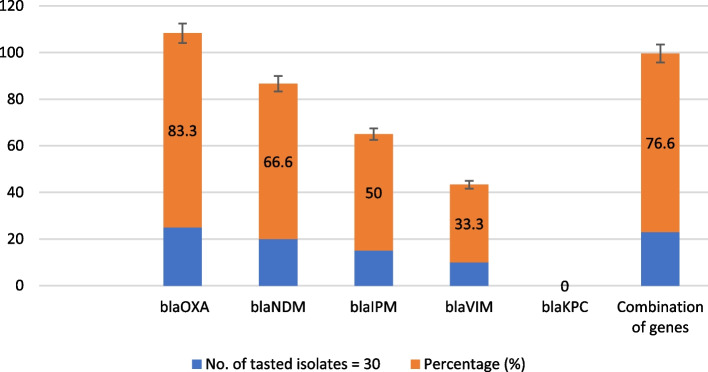
Frequencies and distribution of carbapenemase genes in *P. aeruginosa* isolates

**Fig. 3:**
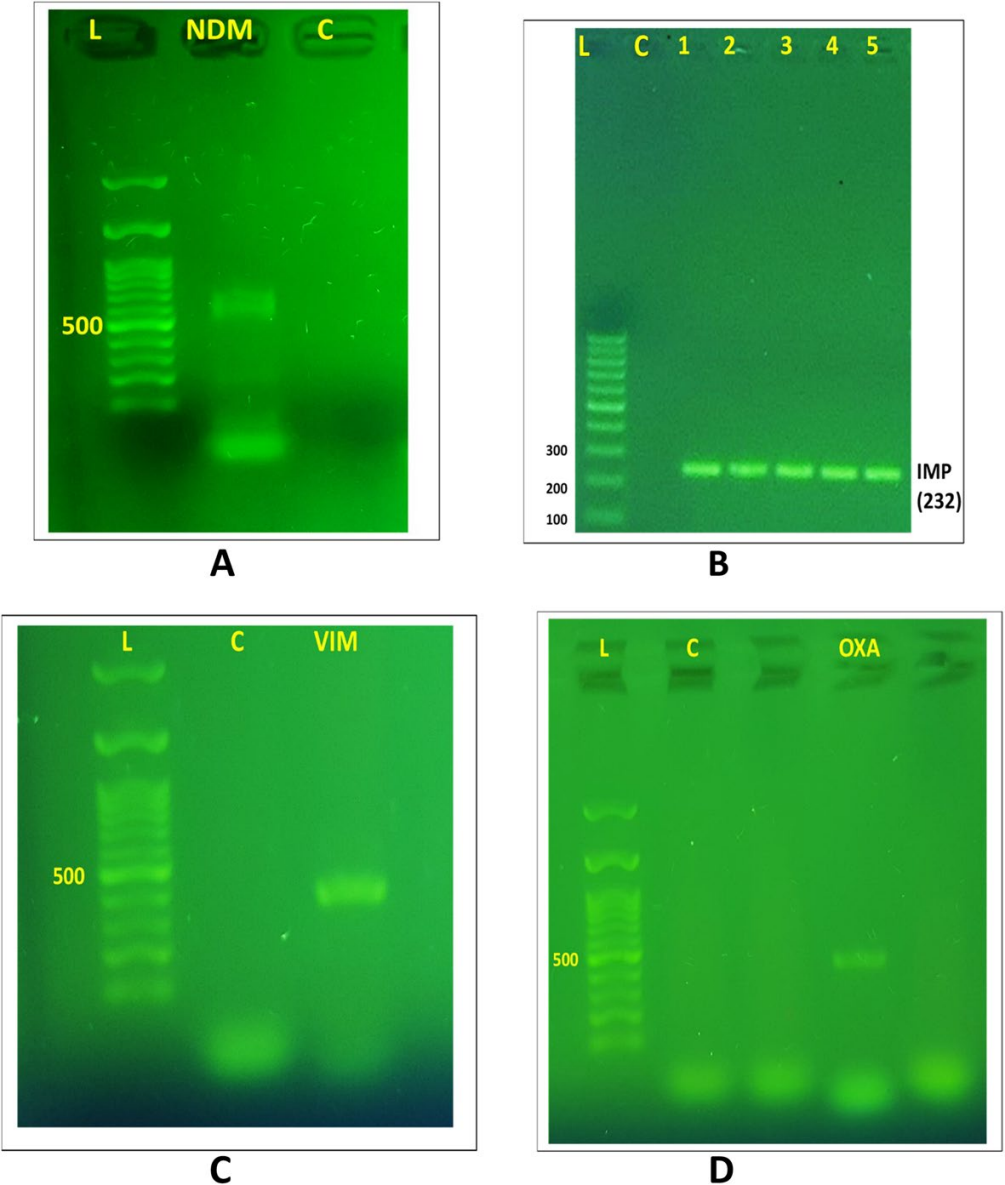
Detection of carbapenemase genes IMP, and VIM in carbapenemase-producing *P. aeruginosa* isolates. Lane M represents a 1-kb DNA ladder and lane C is a negative control. **A** The gel electrophoresis displayed the presence of carbapenemase gene NDM (621 bp). **B **Lanes 1–5 represent the positive IMP carbapenemase gene. **C** Gel electrophoresis revealed the presence of *bla*_VIM_ (390 bp). **D** The gel electrophoresis showed the presence of the carbapenemase gene *bla*_OXA-48_ (438 bp)

The MDR profile, increased incidence, and rapid dissemination of carbapenemases across species through transmissible genetic factors make them the most urgent resistance problem in Gram-negative bacteria [41, 42]. These genes often carry other antimicrobial resistance genes, leading to MDR or extensively drug-resistant traits, limiting treatment options and posing a triple threat. Figure 2 displays the prevalence of carbapenemase genes in 30 isolates that were tested. A study found that all 30 *P. aeruginosa* isolates tested had one or more carbapenemase genes. The most common carbapenemase gene was *bla*_OXA-48_, present in 83.3% of isolates. Other common carbapenemase genes included *bla*_*NDM*_ (66.6%), *bla*_IMP_ (50%), and *bla*_VIM_ (33.3%), and none of the isolates tested positive for *bla*_KPC_ (Fig. 3 A, B, C, and D). 76.6% of positive isolates had multiple carbapenemase genes, ranging from two to four. Statistical analysis showed a significant difference (*p* < 0.001) in carbapenemase resistance genes prevalence among MDR-*P. aeruginosa* isolates.

This study found that *bla*_OXA-48_ is the most common carbapenemase gene, followed by *bla*_NDM._ Subramaniyan and Meenakshi's found that 13.1% of MBL-producing *P. aeruginosa* strains from the ICU were 13.1% *bla*_VIM-4_, 9.8% *bla*_VIM–5_, and 3.2% *bla*_VIM–38_, with none testing positive for KPC, NDM, and IMP genes [31]. Sheikh et al. identified 236 carbapenem-resistant *P. aeruginosa* isolates, with 116 carrying MBL genes and 29 testing positive for *bla*_NDM-1_ [35]. Similarly, Ramadan et al. found that genes encoding VIM, GES, NDM, and IMP were detected in 50%, 40.9%, 27.3%, and 18.2% of isolates [8]. None of the tested isolates showed the presence of the *bla*_*KPC*_ gene, as indicated by earlier research findings [8, 35]. This could be attributed to the existence of additional class A carbapenemases [43]. OXA-48, a highly prevalent worldwide ailment, has reached epidemic proportions in multiple Mediterranean nations, such as Turkey, Iran, Morocco, and Lebanon [35, 44]. The study found that a significant proportion of isolates (76.6%) carried multiple carbapenemase genes, indicating their ability to produce MDR determinants. This is a major concern for antibacterial treatment strategies, as it has been reported in other studies [1, 35].

### Biosynthesis of TiO_2_NPs

The study examined all isolates for TiO_2_NP production, but only one *P. aeruginosa* isolate demonstrated the ability to synthesize TiO_2_NPs.

### Characterization of TiO_2_NPs:

**Fig. 4:**
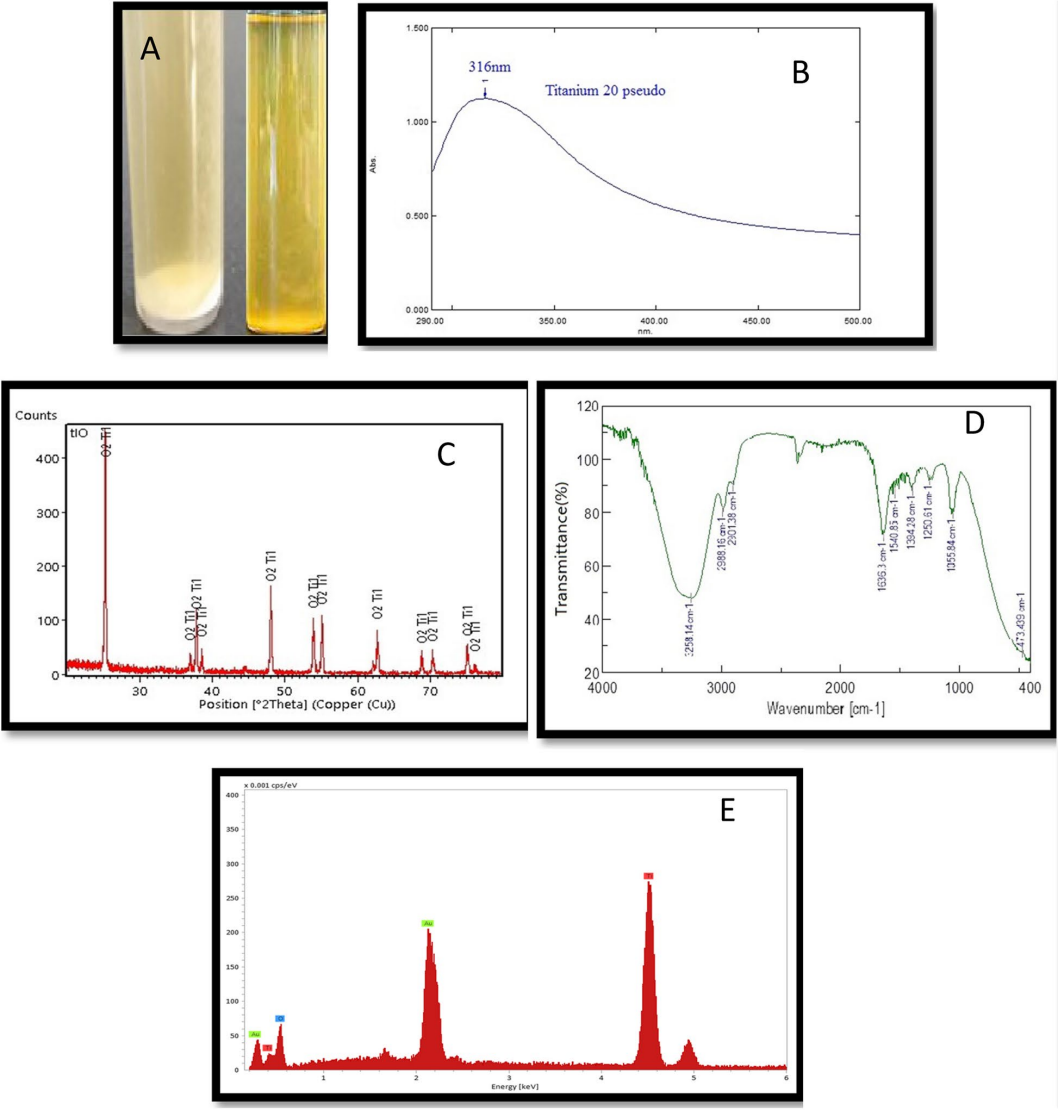
The positive result of color change and UV–visible spectroscopy of biosynthesized TiO_2_NPs (**A **and** B**), XRD results (**C**), FT-IR spectra analysis (**D**), and EDX analysis (**E**)
- Microbial systems, including bacteria, are efficient in producing reducing components for nanomaterial synthesis due to richness in several biomolecules can transform the Ti salts to TiO_2_NPs [46]. *P. aeruginosa* was chosen for TiO_2_ synthesis due to its environmental compatibility, low energy consumption, and cost-effectiveness [47].
- 2- X-ray diffraction analysis
  The synthesized nanoparticles' chemical composition and crystalline properties were verified using X-ray diffraction (XRD) analysis [21]. The crystal structure of TiO_2_NPs was examined using an X-ray diffractometer, which confirmed the formation of TiO_2_NPs from *P. aeruginosa* culture supernatant, with XRD patterns matching standard diffraction data (JCPDS card number: 98–017-2916). The XRD analysis of synthesized TiO_2_NPs revealed distinct diffraction peaks at various angles, including 25.30°, 36.96°, 37.81°, 38.56°, 48.04°, 53.88°, 55.07°, 62.68°, 70.31°, 75.09°, and 76.09°, corresponding to the crystal planes 101, 103, 004, 112, 200, 105, 211, 204, 220, 215, and 224, respectively (Fig. 4 C). The synthesized sample has a small size, crystalline structure, and high purity, as indicated by sharp diffraction patterns [26]. The XRD results from Fig. 4 C indicate that the nanoparticle structure primarily consists of the anatase crystalline phase, known for its high photocatalytic activity, and the main peaks' positions align with previous literature findings [26, 48].
- 3-Fourier Transform Infrared Spectrometry (FTIR)
  Fourier transform infrared (FTIR) spectroscopic analysis was conducted to investigate the interaction between the NPs and capping agents. The FTIR spectra were recorded in the wavenumber range of 400–4000 cm-1. The FTIR spectra of TiO_2_NPs synthesized by *P. aeruginosa* exhibited prominent peaks at 3258.14, 2988.16, 2901.38, 1636.3, 1540.85, 1394.28, 1250.61, 1055.84, and 473.43 cm − 1 (Fig. 4 D). The biosynthesized TiO_2_NPs showed a broad band at 3258 cm − 1, indicating O–H stretching due to the alcoholic group. The peaks at 2988 cm − 1 and 2901 cm − 1 corresponded to the C–H of CH2 and CH3 groups in aliphatic chains, while signals at 1636 cm − 1 and 1540 cm − 1 indicated the presence of amide and amine groups.
- The FTIR analysis was used to investigate the reduction of TiO_2_NPs by biomolecules in microbial cells. The peaks at 1394 cm − 1 and 1250 cm − 1 were attributed to C-O stretch vibrations, possibly indicating an alcohol or carboxylic acid group. The bands at 1055 cm − 1 and 473 cm − 1 represented C-O stretching vibrations of aliphatic amines and Ti–O stretching vibrations respectively. These biomolecules may also play a role in nucleation and biosynthesis processes, acting as stabilizing and capping agents [49, 50].
- 4- Energy-dispersive spectroscopic analysis (EDX)
- The energy-dispersive spectroscopic analysis confirmed the elemental distribution of Ti and O elements, confirming the formation of TiO_2_ (Fig. 4 E), in line with previous studies.
- 5- Morphology and shape of TiO_2_NPs
  - 1-Transmission *electron* microscopy (TEM)
    The study analyzed *P. aeruginosa* nanoparticles using TEM to determine their size, morphology, and distribution at the nanoscale. The TiO_2_NPs were found to be predominantly spherical and ellipsoidal, with uneven distribution Fig. 5 A. The average diameters and size distributions were determined using ImageJ software, with the majority falling within the 30–40 nm range, accounting for 18% of the distribution (Fig. 5 C). The study's findings, compared to previous studies by Khan, Fulekar, and Eisa et al. [22, 51], were found to align with the literature, with a size distribution histogram indicating sample polydispersity, a common characteristic of nanoparticles synthesized through biosynthetic methods [52]. The study found that most TiO_2_NPs were spherical or round to ellipsoidal in shape, with both individual NPs and agglomerates observed (Fig. 5 A). The nanoparticles appeared separated from each other, suggesting spiraling peptides around them.

- 1-visual observation and UV–visible analysis
  The stability and formation of synthesized TiO_2_NPs in an aqueous colloidal solution were determined through visual observation and UV–visible analysis [21]. The isolated *P. aeruginosa* strain successfully biosynthesised TiO_2_NPs by exposing bacterial cultures to TiO_2_, which was confirmed by the color changes from yellow to white precipitate, indicating the synthesis of TiO_2_NPs (Fig. 4 A). The color generation in TiO_2_NPs is due to the excitation of surface plasmon in metal nanoparticles [45]. UV absorption analysis confirmed the synthesis, with a clear absorption peak indicating the anatase phase of nano-TiO_2_ (Fig. 4 B). The cut-off wavelength was 316 nm, consistent with previous research on TiO_2_NP synthesis using various bacterial strains.

**Fig. 5:**
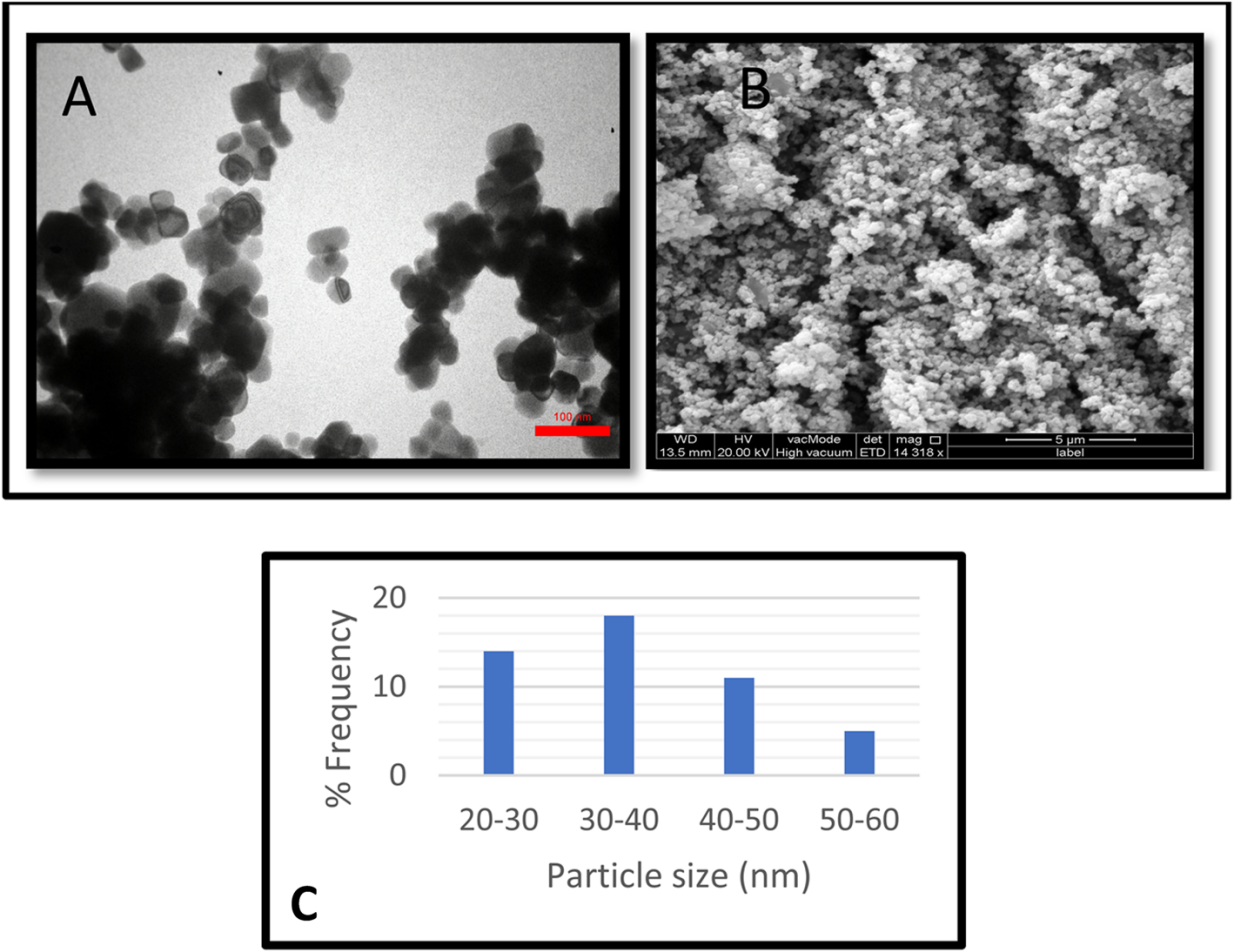
(**A**) TEM analysis and (**B**) FE-SEM analysis of biosynthesized TiO_2_NPs (**C**) Histogram of the particle size distribution of the biosynthesized TiO_2_NPs
  - 2- Field emission scanning *electron* microscopy (FESEM)
    The surface morphology and shape of synthesized TiO_2_NPs were analyzed using SEM, with the results showing uniform spherical shapes and dimensions. This aligns with previous studies [26, 28]. Additionally, the results of Mathesh et al. showed that Spirulina-mediated TiO_2_ nanoparticles were efficient against multidrug-resistant bacteria due to their spherical form with aggregation in SEM imaging and XRD analysis, which showed 61.4% crystallinity with anatase phase [53]. The SEM images are shown in Fig. 5 B.

### The antibacterial efficacy of TiO_2_NPs

**Fig. 6:**
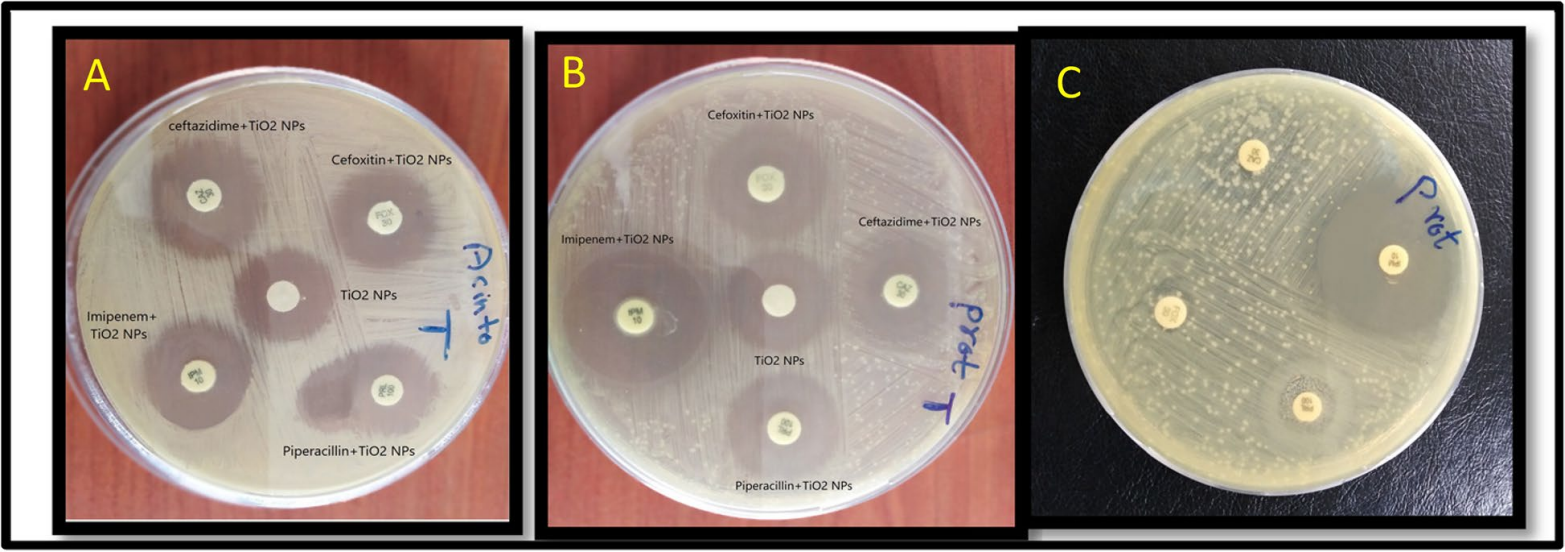
The combined effect between antibiotics and biosynthesized TiO_2_NPs against *Proteus sp.* (**A**), *Acinetobacter baumannii* (**B**)**,** and Antibiotic resistance in *Proteus sp.* (**C**)

Table 3 presents the disc diffusion method results for evaluating the effects of TiO_2_NPs with and without different β-lactam antibiotic combinations, on MDR strains of *E. coli, Klebsiella sp., A. baumannii, Proteus sp.,* and *P. aeruginosa* (Table 3). The bacteria were tested against four antibiotics: imipenem, ceftazidime, piperacillin, and cefoxitin. The results showed that all tested antibiotics were ineffective against the bacteria, which exhibited resistance to these antibiotics, according to the Clinical and Laboratory Standards Institute (2020) (Table 3 and Fig. 6 C).

**Table 3:** Mean inhibition (mm) and fold area increase of different antibiotics, TiO_2_NPs, and combined against MDR bacteria. The test is repeated 3 times. *p < 0.01

Antibiotics (μg/disc)	*E. coli*			*Klebsiella sp.*			*Pseudomonas aeruginosa*			*Acinetobacter baumannii*			*Proteus sp.*		
	**Antibiotics**	**Antibiotics + TiO**_**2**_** NPs**	**FA %**	**Antibiotics**	**Antibiotics + TiO**_**2**_** NPs**	**FA %**	**Antibiotics**	**Antibiotics + TiO**_**2**_**NPs**	**FA %**	**Antibiotics**	**Antibiotics + TiO**_**2**_**NPs**	**FA %**	**Antibiotics**	**Antibiotics + TiO**_**2**_** NPs**	**FA%**
**Imipenem**	12	25	108	13	25	92	10	24	140	9	25	177	11	27	145
**Ceftazidime**	8	22	175	9	23	155	8	26	225	10	26	160	8	23	187
**Piperacillin**	6	21	250	8	24	200	6	22	266	6	23	283	6	23	283
**Cefoxitin**	6	20	233	6	22	266	9	27	200	8	24	200	6	22	266
**TiO**_**2**_** NPs**	18			20			19			22			21		
	*P*-value < 0.01														

*FA* Fold area increasing in the antibiotics

The biosynthesized TiO_2_NPs, at a concentration of 5000 μg/mL, demonstrated a significant (*P* < 0.01) antibacterial effect against MDR *A. baumannii* and *Proteus* sp. strains. The study found that *A. baumannii* and *Proteus* sp. showed the largest zone of inhibition (22 mm and 21 mm, respectively), while *E. coli, Klebsiella sp.,* and *P. aeruginosa* had zones of inhibition of 18 mm, 20 mm, and 19 mm, respectively. The study found that TiO_2_NPs were more effective against MDR Gram-negative bacilli strains than all tested antibiotics due to their resistance to antibiotic discs (Table 3 and Fig. 6 A and B).

TiO_2_NPs are a type of metal oxide nanoparticles that have been extensively studied for their antimicrobial properties. They have been found to possess the potential to eradicate both Gram-positive and Gram-negative bacteria [16, 45]. When compared to antibiotics alone, TiO_2_NPs demonstrated the strongest antibacterial efficacy against manty pathogens [54]. Studies have shown that *P. aeruginosa* synthesizes TiO_2_NPs with antibacterial activity, and similar findings have been observed against other bacteria [51, 55–57]. A study in Iraq [58] found that TiO_2_NPs at a concentration of 500 μg/mL showed the highest zone of inhibition against MDR test organisms, with a maximum of 24 mm against *Streptococcus pyogenes*, showing high sensitivity even at 31.25 μg/mL. *Proteus vulgaris* showed the least sensitivity. Gram-negative bacteria have higher negative charges and stronger adhesion to positive surfaces, making biosynthesized TiO_2_NPs more effective against them, regardless of resistance level. It is possible that oxidation and cell death are brought on by the electromagnetic attraction between bacteria and metal oxides. [59]. The bactericidal effect of tested nanoparticles may also be due to reactive oxygen species (ROS), primarily hydroxyl radicals (OH), decomposing bacterial outer membranes, leading to phospholipid peroxidation and oxidative cell death [51]. In addition to rupturing cell membranes, the main role of cytoplasmic ROS generation in NPs' antibacterial actions is to seriously damage bacteria's DNA, which eventually results in the bacterial demise. Furthermore, interactions between NPs and phosphate components within the bacterial cytoplasm may result in the formation of persistent complexes that disrupt essential bacterial enzymes [54, 60].

### Synergistic effect of TiO_2_NPs with antibiotics by disc diffusion methods

The study examined the effects of TiO_2_NPs and four β-lactam antibiotics [imipenem (10 μg), ceftazidime (30 μg), piperacillin (100 μg), and cefoxitin (30 μg)] against MDR-Gram-negative bacteria, including *E. coli, Klebsiella sp., P. aeruginosa, A. baumannii,* and *Proteus sp.* The disc diffusion method was used to measure the inhibition zones of antibiotics alone and in combination with TiO_2_NPs at a concentration of 5000 μg/mL [29]. Table 3 shows that the inhibition zone diameter increased when tested antibiotics with TiO_2_NPs at 5000 μg/mL concentrations against tested isolates. The study measured the inhibition zone produced by antibiotics before and after treatment with TiO_2_NPs (Table 3 and Fig. 6 A and B), revealing a significant (P < 0.01) increase in the fold area. especially, the zone of inhibition for piperacillin was 6 mm before nanoparticle treatment, and 23 mm after treatment against *Proteus sp.* and *A. baumannii*. The combination of antibiotics and TiO_2_NPs shows synergistic effects, with imipenem showing the lowest antibacterial activity increase in area (13 mm to 25 mm) against *Klebsiella sp*. The study evaluated the antibacterial activity of antibiotic discs and TiO_2_NPs in combination with all MDR bacterial isolates. The most significant increase areas (283%) were observed in piperacillin against *Proteus sp.* and *A. baumannii*, as well as cefoxitin (266%) against *Klebsiella sp*. and *Proteus sp.* (Table 3 and Fig. 6. A and B). The increase in surface areas of inhibition zones varied from 92% up to the considerable amount of 283% and 266% in the presence of TiO_2_NPs (Table 3). TiO_2_NPs showed the most significant antibacterial effect against *Proteus sp., A. baumannii*, in addition, cefoxitin, and their combination with all antibiotics showed increased antibacterial activity compared to individual antibiotics. The combination therapy of TiO_2_NPs with antibiotics, results in a significant increase in antibacterial activity, possibly by synergistically targeting different bacterial cellular processes and pathways, overcoming antibiotic resistance mechanisms [60]. The rise in infectious diseases associated with antibiotic-resistant pathogenic strains has necessitated the search for alternative antibiotics with potent bactericidal and bacteriostatic properties. Microorganisms have developed complex resistance mechanisms, such as inactivation of antibiotics, alteration of target sites, and transformation of metabolic pathways, due to repeated exposure to antibiotics over generations [61]. Arora et al.'s study found that ceftazidime, an antibiotic, showed a significant synergistic effect (24 mm zone of inhibition) when combined with TiO_2_NPs, but its antibacterial activity did not improve when used with cefotaxime against MDR-*Pseudomonas sp.* [62]. Previous studies have shown that TiO_2_NPs when combined with 23 commercial antibiotics effectively combat MRSA clinical isolates. The combination showed higher activity than individual antibiotics, with amikacin and penicillin G showing the greatest increase in efficacy among tested antibiotics [63]. Because of the active ions, nanoparticles have an incontrovertible antibacterial action and minimal harm to human cells. Additionally, it is a persistent biocide with low volatility and good thermal stability [64]. Hassan et al.'s study found that TiO_2_NPs mediated by *H. lbiecea* showed greater bacterial growth inhibition than commercial TiO_2_NPs against *Staphylococcus aureus*, *E. coli*, and *Bacillus subtilis*, with zones of inhibition of 10.5 mm, 17 mm, and 15 mm respectively [21]. Functionalization of nanoparticles with antibiotics offers a promising platform to combat bacterial resistance, reduce drug dosage and toxicity, and specifically target infection sites, overcoming resistance and minimizing harm to normal cells [65]. TiO_2_NPs are gaining attention in health and industry due to their photocatalytic properties, low toxicity, and absence of allergic reactions, influenced by factors like particle size and synthesis method [47].

### Antibiofilm activity of TiO_2_NPs

**Fig. 7:**
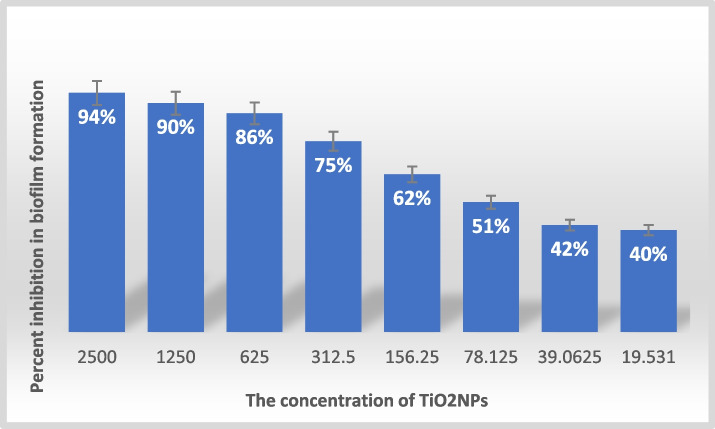
The antibiofilm inhibition percentage of biosynthesized TiO_2_NPs on strong biofilm-producing*-P. aeruginosa*

The National Institutes of Health and Center for Disease Control report that 65–80% of infections are caused by microbes that form biofilms, with the most common being *P. aeruginosa, E. coli*, and *Staphylococcus aureus* [66]. The study reveals that biosynthesized TiO_2_NPs have antibiofilm properties against 25 MDR-*P. aeruginosa* strains, effectively inhibiting the formation of biofilms. The study revealed that TiO_2_NPs effectively inhibited the attachment and formation of biofilms by tested *P. aeruginosa* strains in a dose-dependent manner. The study found that biosynthesized TiO_2_NPs significantly (*p* < 0.001) reduced the attachment of strong biofilm-producing *P. aeruginosa* compared to the control group. Treatments with different concentrations of TiO_2_NPs (19.531, 39.0625, 78.125, 156.25, 312.5, 625, 1250, and 2500 μg/ml) reduced biofilm activity by 40%, 42%, 51%, 62%, 75%, 86%, 90%, and 94% compared to control cultures without TiO_2_NPs (Fig. 7).

**Fig. 8:**
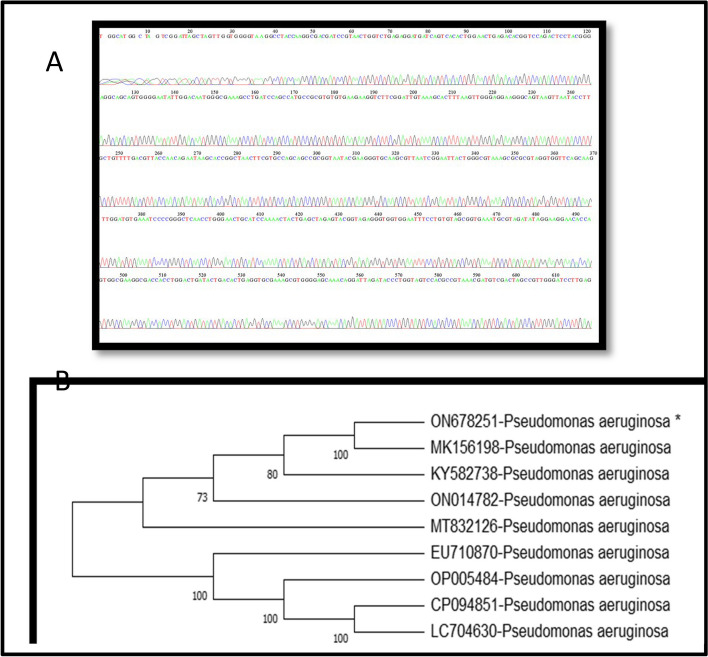
A displays complete 16S rRNA gene nucleotide sequences for *P. aeruginosa*, from a newly obtained isolate from Erbil-Kurdistan Region-Iraq, assigned NCBI GenBank accession number ON678251. Peak of sequencing of 16S rRNA gene of *P. aeruginosa* (**A**). Phylogenetic neighbor- joining trees of 16S rRNA amplified from the tested isolates of *P. aeruginosa*. The bacteria recovered in the present study are marked with an asterisk (**B**)

TiO_2_NPs disrupt biofilm formation by potentially interfere with quorum sensing mechanisms in bacteria, which are essential for biofilm formation and communication among bacterial cells. By disrupting these pathways interacting with biofilm matrix components, leading to destabilization and inhibition of biofilm development [67]. The mechanism of action of TiO_2_NPs involves targeting sulfhydryl groups in the cell membrane, forming S-TiO_2_ bonds, and suppressing the electron transport chain and enzymes essential for biofilm formation [13]. Abdulazeem et al.'s study on bacterial biofilm eradication using TiO_2_NPs revealed that treatment with MIC concentrations reduced biofilm growth compared to a control group without NPs [9]. Landage et al. found that TiO_2_NPs reduced biofilm formation by 40–50% in both Gram-negative and Gram-positive bacterial strains, particularly in *P. aeruginosa* [26]. Alavi & Karimi's study found that Ag-TiO_2_ nanocomposites reduced biofilm roughness, a virulence factor linked to quorum sensing mechanisms in *P. aeruginosa* ATCC 27853 [68].The fusion of nanoparticles with the bacterial cell membrane is what causes the significant antimicrobial effects of nanoparticles, which cease bacteria from growing to the point where they can form a biofilm. This is another reasons for the increased anti-biofilm effects of nanoparticles [69].

### TiO_2_NP-producing *P. aeruginosa* isolate's 16S rRNA gene sequence and phylogenetic analysis

The BLAST alignment program confirmed the isolate's alignment agreement, revealing that the 16S rRNA sequence of *P. aeruginosa* shares 100% homology with strains MK156198, KY582738, MT832126, ON014782, and MZ191664 from Iraq, Egypt, and Singapore, respectively. The 16S rRNA gene sequencing of a *P. aeruginosa* isolate showed high homology with other local and global strains, with a range of 100% to 99.89% (Table 4 and Fig. 8. B).

**Table 4:** Percentage distribution of *P. aeruginosa* according to blast of GenBank NCBI of partial 16S rRNA gene

Bacteria sample Accession Number	Query Cover %	Identic Number %	GenBank Accession Number	GenBank Species Identification	Country
**ON678251**	100	100	MK156198	*Pseudomonas aeruginosa*	Iraq
	100	100	KY582738	*Pseudomonas aeruginosa*	Iraq
	100	100	ON014782	*Pseudomonas aeruginosa*	Egypt
	100	100	MZ191664	*Pseudomonas aeruginosa*	Singapore
	100	100	MT832126	*Pseudomonas aeruginosa*	Iraq
	100	99.89	EU710870	*Pseudomonas aeruginosa*	Japan
	100	99.89	OP005484	*Pseudomonas aeruginosa*	China
	100	99.89	CP094851	*Pseudomonas aeruginosa*	China
	100	99.89	LC704630	*Pseudomonas aeruginosa*	Nigeria

This homology was observed with *P. aeruginosa* strains from China, Japan, and Nigeria, and is consistent with other *P. aeruginosa* isolates in the GenBank database [30].

## Conclusions

Multi-drug resistant *P. aeruginosa* isolates require complex therapy strategies due to their high resistance to a variety of antimicrobial agents. Precise detection of bacterial infections resistant to carbapenem is essential for managing patient care and preventing contamination. The results of this study demonstrate the effectiveness of successfully biosynthesized TiO_2_NPs. The combination of antibiotics and biosynthesized TiO_2_NPs demonstrated potent antibacterial and anti-biofilm effects particularly for controlling MDR- *P. aeruginosa*. This combination therapy can effectively eliminate the pathogen's antibiotic resistance and biofilm formation, posing a significant global public health threat. The synergistic activity of biosynthesized TiO_2_NPs with antibiotics presents a valuable warrant for further investigation in clinical applications.

## Supplementary Information

Supplementary Material 1.

## Data Availability

The datasets generated and/or analysed during the current study are available in the [TiO2NP-producing P. aeruginosa isolate's 16S rRNA gene sequence] repository, [https://data.mendeley.com/datasets/8xxfgwt4z6/1]. The nucleotide sequence of the P. aeruginosa strain was submitted to the National Centre for Biotechnology Information (www.ncbi.nlm.nih.gov), assigned GenBank accession number ON678251.

## Abbreviations

*A. baumannii*: *Acinetobacter baumannii*
*E. coli*: *Escherichia coli*
FE-SEM: Field Emission Scanning Electron Microscopy
FTIR: Fourier-Transform Infrared Spectroscopy
*K. pneumonia*: *Klebsiella pneumonia*
MBL: Metallo-beta-lactamase
MDR: Multidrug-Resistant
NP: Nanoparticles
OD: Optical density
*P. aeruginosa*: *Pseudomonas aeruginosa*
PCR: Polymerase Chain Reaction
S-TiO_2_ bonds: Sulfhydryl-TiO_2_ Bonds
TEM: Transmission Electron Microscopy
TiO_2_NPs: Titanium Dioxide Nanoparticles
TSB: Tryptic Soy Broth
XRD: X-ray Diffraction
