# Supplementary figures and images for: The enhanced antibacterial and antibiofilm properties of titanium dioxide nanoparticles biosynthesized by multidrug-resistant Pseudomonas aeruginosa
Source: BMC Microbiol. 2024 Oct 1;24:379. doi: 10.1186/s12866-024-03530-y (PMC11443863; doi:10.1186/s12866-024-03530-y)

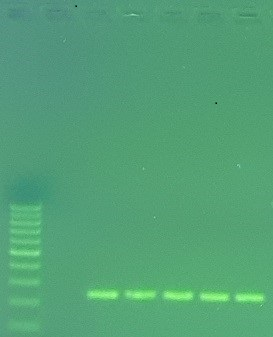

Supplement: Supplementary file 1 — Supplementary Material 1. [file 12866_2024_3530_MOESM1_ESM.zip › imp.tiff]

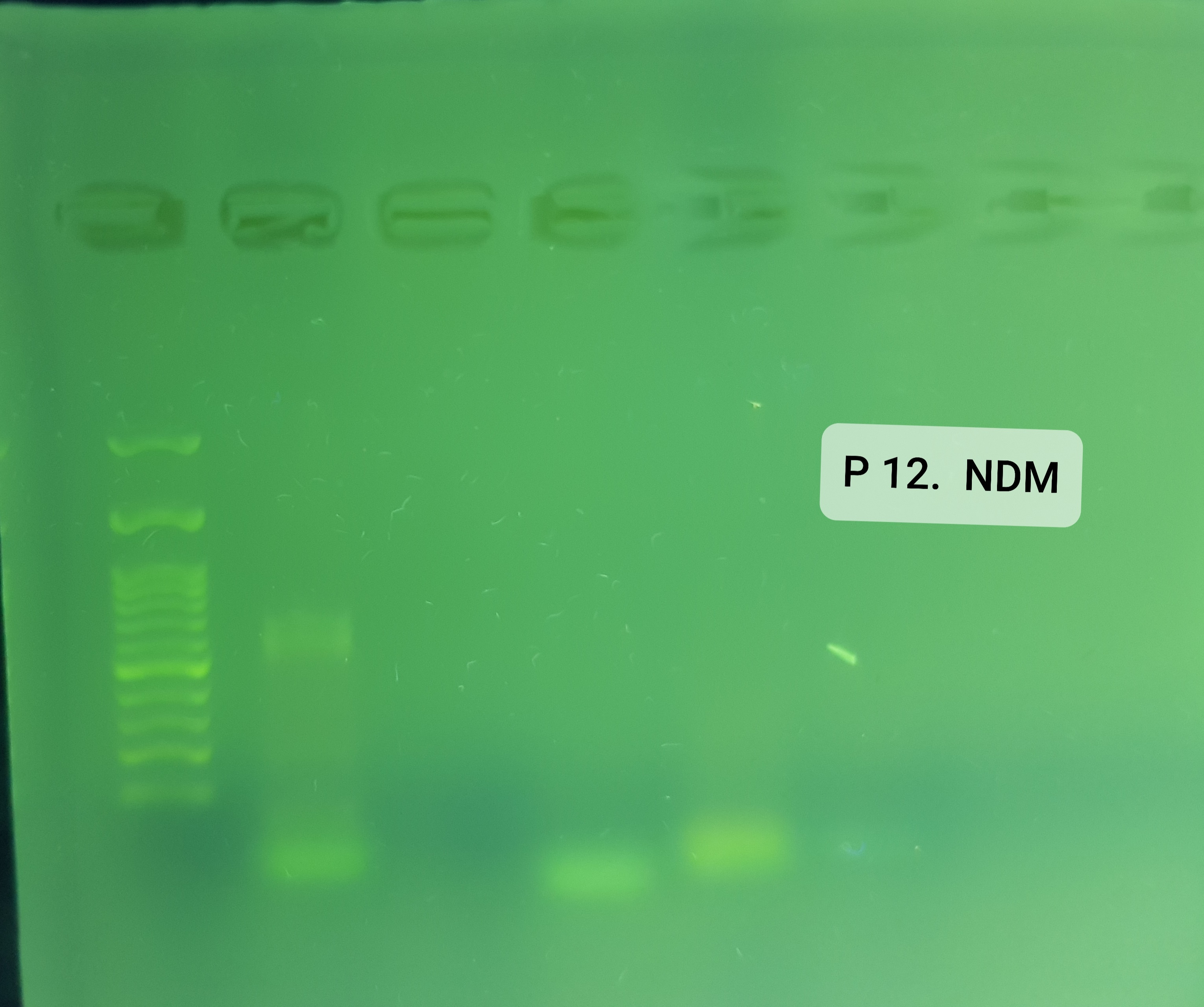

Supplement: Supplementary file 1 — Supplementary Material 1. [file 12866_2024_3530_MOESM1_ESM.zip › ndm.tiff]

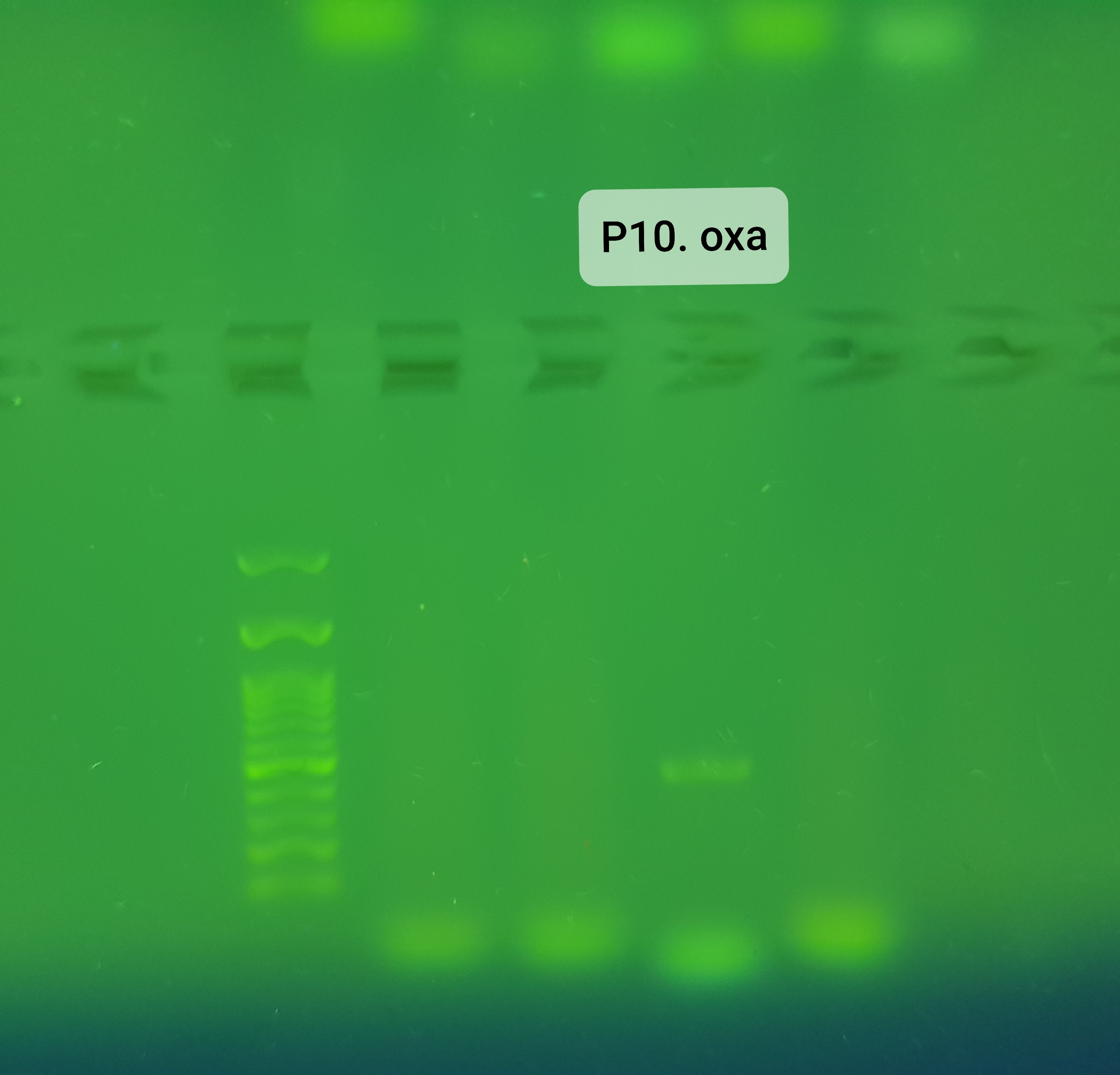

Supplement: Supplementary file 1 — Supplementary Material 1. [file 12866_2024_3530_MOESM1_ESM.zip › OXA.tiff]

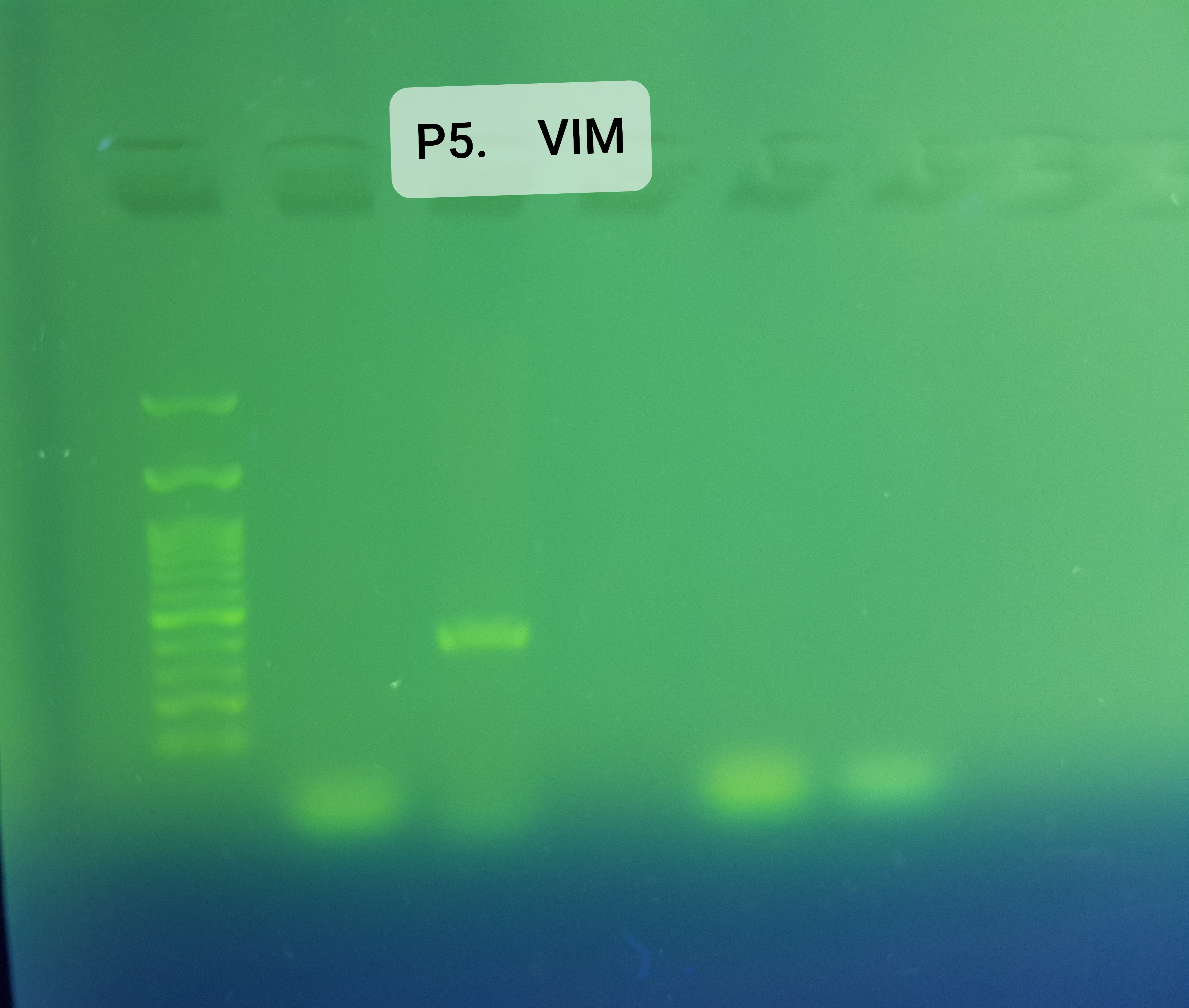

Supplement: Supplementary file 1 — Supplementary Material 1. [file 12866_2024_3530_MOESM1_ESM.zip › VIM.tiff]
